# The Genome of Opium Poppy Reveals Evolutionary History of Morphinan Pathway

**DOI:** 10.1016/j.gpb.2018.09.002

**Published:** 2018-09-28

**Authors:** Yiheng Hu, Ran Zhao, Peng Xu, Yuannian Jiao

**Affiliations:** 1 State Key Laboratory of Systematic and Evolutionary Botany, Institute of Botany, Chinese Academy of Sciences, Beijing 100093, China; 2 University of Chinese Academy of Sciences, Beijing 100049, China

Plants, as primary producers, have been playing an indispensable role in other organisms’ survival and the balance of whole ecosystem on Earth. Especially, they provide the main source of energy, food, and medicine for human beings, some of which are derived from the primary or secondary metabolites [1]. Angiosperms, with more than 300,000 species on Earth, are the largest group of land plants by far. Most agricultural crops, fruits, ornamental plants, and medicinal herbs belong to this group. The medicinal herbs are usually rich in specialized metabolites that could provide safe and valuable resources for pharmaceutical development.

Opium poppy (*Papaver somniferum* L.) is an ornamental and medicinal plant that possesses indispensable medicinal value. It produces various alkaloids including morphinan and noscapine, which are widely used in pain relief and cough suppression. However, it also can cause euphoria, sleepiness, and addiction as side effects [2]. Currently, it remains a large challenge to artificially synthesize substitutes of any morphinan subclass of benzylisoquinoline alkaloids (BIAs) [2]. Deciphering the genome of opium poppy will help understand the molecular mechanism of morphine production.

Whole-genome sequencing has been widely used to decode the gene content and thereafter the biosynthetic pathway of metabolites. For instance, Badouin et al. [3] completed sunflower genome sequencing and combined quantitative genetics and transcriptome data to investigate the candidate genes involved in networks for oil metabolism and flowering time. Similarly, Shang et al. [4] employed a genome-wide association study together with metabolomics to identify nine genes in the cucurbitacin C (CuC) biosynthetic pathway, and successfully discovered two transcription factors controlling the bitterness of cucumber.

Recently, Guo et al. [2] firstly reported the reference genome of opium poppy, which has a large complex genome with over 70% of repetitive sequences (Figure 1A). The final assembled genome size is 2.72 Gb, with annotation of 51,213 protein-coding genes and 9494 non-coding RNAs. They firstly generated two independent *de novo* assemblies based on 10× next-generation and PacBio third-generation sequencing platforms, and then developed a new pipeline to merge the two assemblies, to achieve a high level of sequence continuity. Longer reads from PacBio sequencing were used to elongate scaffolds spanning repeat-rich genomic regions for quality control (QC) purposes. They used PacBio sequencing reads to improve genomic region harboring highly repetitive sequences. Actually, using even longer sequencing reads from Oxford Nanopore Technologies (ONT), they were able to correct a 25-kb missing region in a highly repetitive genomic region of 227 kb in length, where the *T6ODM* gene family coding for thebaine 6-*O*-demethylase is located. Therefore, the genome assembly approaches developed in Guo et al. [2] could be used as an effective strategy to decipher other large and complex plant genomes.

**Figure 1:**
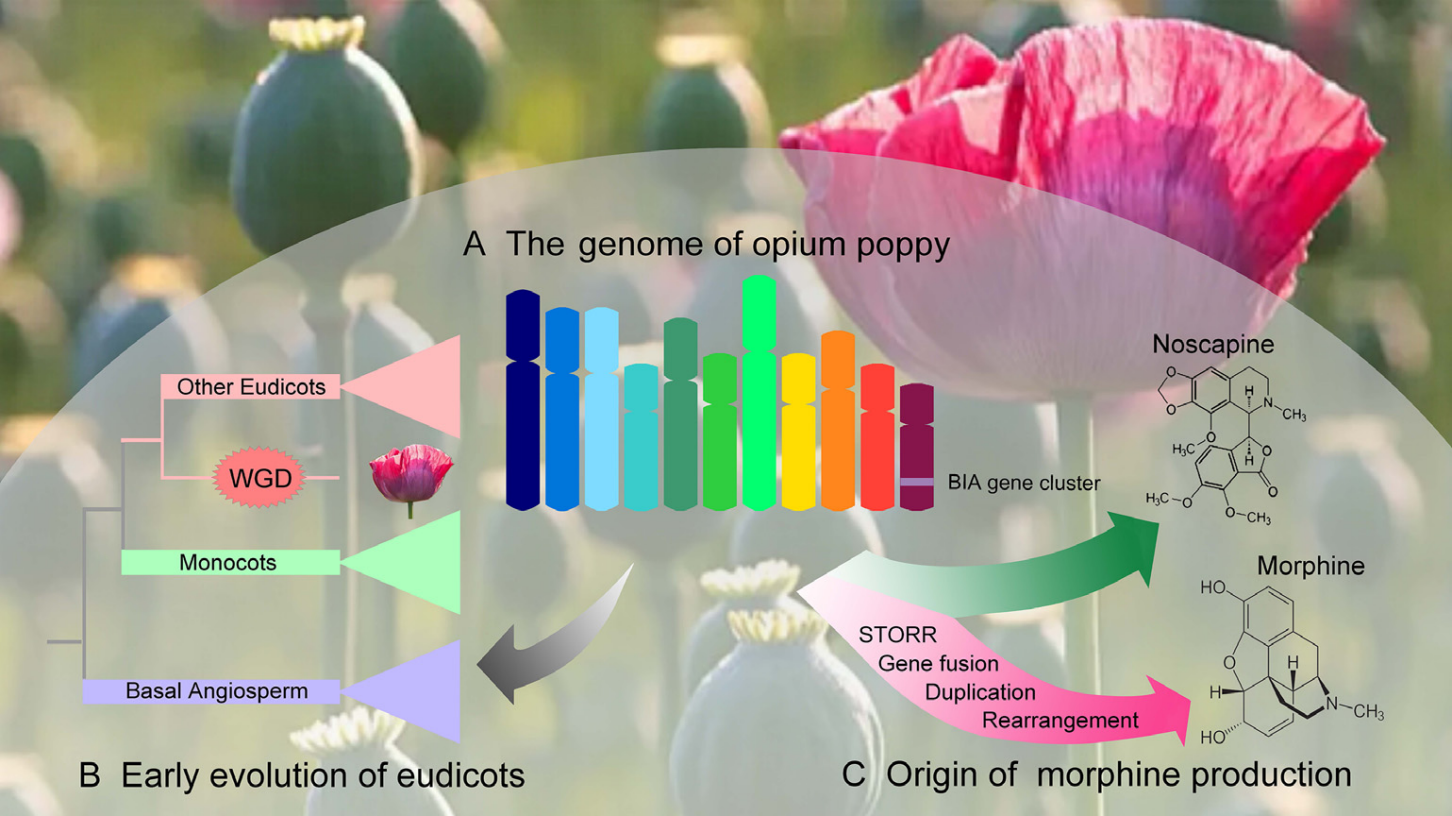
**The opium poppy genome contributes to deciphering early evolutionary history of eudicots and to improving the medicinal poppy** **A.** The high quality genome sequences of opium poppy were assembled to construct 11 pseudo-chromosomes. **B.** As a basal eudicot, opium poppy is a great reference for exploring early evolutionary history of eudicots and beyond. **C.** Genomic data of opium poppy provide insights into the evolutionary origin of morphinan pathway. WGD, whole-genome duplication; BIA, benzylisoquinoline alkaloid; STORR, (S)- to (R)-reticuline.

As a basal eudicot, the opium poppy genome offers a great opportunity to examine the early evolutionary history of eudicots (Figure 1B). Two other basal eudicot genomes have been available so far, including *Nelumbo nucifera* [5] and *Aquilegia coerulea* [6]. Together, they provide a valuable resource for reconstructing the pan-eudicot genome, and should therefore accelerate comparative genomic analysis of eudicots and monocots. In addition, a major polyploidy event or whole-genome triplication (WGT), *gamma*, occurred early in eudicot evolution, and the rapid radiation of core eudicot lineages that gave rise to nearly 75% of angiosperm species appears to have occurred coincidentally or shortly following the *gamma* triplication event [7]. It has been widely acknowledged that polyploidization has played an important role in plant speciation, adaptation, and diversification [8], [9]. By comparative genomics research of opium poppy and other dicots plants, Guo et al. [2] confirmed that *gamma* event was restricted to core-eudicots clade. Moreover, they also identified an independent whole-genome duplication (WGD), which occurred around 7.8 million years ago (MYA) in opium poppy lineage.

It has been suggested that clusters of functionally-related genes are usually associated with the biosynthesis of certain metabolites [10]. The well-assembled opium poppy genome makes it easier to locate all related genes of BIA metabolism and investigate their evolutionary history. Guo et al. [2] found that BIA gene cluster, spanning a genomic region of 584 kb on chromosome 11, contributes to the noscapine and morphine biosynthesis. By employing syntenic analysis, they found that part of the BIA gene cluster involved in noscapine branch is assembled by an ancient duplication event. The other genes, except *STORR* encoding (S)- to (R)-reticuline, in the BIA gene cluster that are involved in the morphinan branch pathway are likely recruited from the recent WGD around 7.8 MYA [2]. Therefore, multiple gene duplication events played an important role in the evolutionary history of the BIA gene cluster in opium poppy (Figure 1C).

In addition, Guo et al. [2] elucidated the evolutionary history of the *STORR* gene, resulting from an gene fusion event, which is key to the origin of morphinan biosynthesis in Papaveraceae. Following a duplication event before the WGD (∼7.8 MYA), the genes encoding P450 and oxidoreductase underwent an 865-bp deletion leading to the *STORR* gene fusion. Additionally, Guo et al. also investigated the copy number, genomic location, and sequence identity of three key enzymes in morphinan pathway, including T6ODM, codeine 3-*O*-demethylase (CODM), and codeinone reductase (COR) [2]. Specifically, the BIA gene cluster was assembled before the independent WGD event and further duplicated after the WGD, and the *STORR* gene fusion is key to morphinan biosynthesis in opium poppy. The synergistic and efficient synthesis of the special metabolic products in opium poppy can be attributed to the complicated evolutionary history of opium poppy.

In short, Guo et al. [2] developed an effective strategy that could be employed for others to decipher large and complex plant genomes. The genomic data of opium poppy, as an additional reference genome of basal eudicots, provide a valuable resource for future evolutionary studies on eudicots, monocots, as well as comparative genomics within angiosperms. Finally, Guo et al. [2] deciphered the key evolutionary events leading to the origin of morphinan production. Further detailed steps during the formation of the morphinan pathway could be investigated if other genomic information becomes available for closely-related species, which would be of great value for molecular breeding and ultimately for improving the production of morphinan in opium poppy.

## Competing interests

The authors declare that they have no competing interests.

## References

[1] Chen W., Wang W., Peng M., Gong L., Gao Y., Wan J. (2016). Comparative and parallel genome-wide association studies for metabolic and agronomic traits in cereals. Nat Commun.

[2] Guo L., Winzer T., Yang X., Li Y., Ning Z., He Z. (2018). The opium poppy genome and morphinan production. Science.

[3] Badouin H., Gouzy J., Grassa C.J., Murat F., Staton S.E., Cottret L. (2017). The sunflower genome provides insights into oil metabolism, flowering and asterid evolution. Nature.

[4] Shang Y., Ma Y., Zhou Y., Zhang H., Duan L., Chen H. (2014). Biosynthesis, regulation, and domestication of bitterness in cucumber. Science.

[5] Ming R., Vanburen R., Liu Y., Yang M., Han Y., Li L.T. (2013). Genome of the long-living sacred lotus (*Nelumbo nucifera* Gaertn.). Genome Biol.

[6] Filiault D., Ballerini E., Mandakova T., Akoz G., Derieg N., Schmutz J. (2018). The *Aquilegia* genome: adaptive radiation and an extraordinarily polymorphic chromosome with a unique history. bioRxiv.

[7] Jiao Y., Leebensmack J., Ayyampalayam S., Bowers J.E., Mckain M.R., Mcneal J. (2012). A genome triplication associated with early diversification of the core eudicots. Genome Biol.

[8] Van de Peer Y., Mizrachi E., Marchal K. (2017). The evolutionary significance of polyploidy. Nat Rev Genet.

[9] Jiao Y. (2018). Double the genome, double the fun: genome duplications in angiosperms. Mol Plant.

[10] Nützmann H.W., Osbourn A. (2014). Gene clustering in plant specialized metabolism. Curr Opin Biotechnol.

